# Inflammatory markers CRP and WBC as predictors of liver function impairment in multiple injury trauma patients: a repeated measures analysis

**DOI:** 10.3389/fmed.2025.1570474

**Published:** 2025-08-01

**Authors:** Gang Wang, Cui Tang, Rui Wang, Chundao Xu, Tugang Shen, Gaokai Li

**Affiliations:** ^1^School of Life and Health, Huzhou College, Huzhou, Zhejiang, China; ^2^Zhejiang Xinda Hospital, Huzhou, Zhejiang, China; ^3^Department of Orthopaedics, South Taihu Hospital Affiliated to Huzhou College, Huzhou, Zhejiang, China

**Keywords:** multiple trauma, liver function index, inflammatory index, generalized estimating equation, gender difference

## Abstract

**Aims:**

To investigate the relationship between liver function indices (AST, ALT, GGT) and inflammatory markers (CRP, white blood cell count) in multiple injury patients at different time points post-trauma.

**Methods:**

We retrospectively analyzed 114 patients with multiple injuries admitted to a tertiary trauma center in Zhejiang Province (January 2023–December 2024). AST, ALT, GGT, CRP, white blood cell count, NLR, and LMR were measured at admission, 24 h, and 72 h post-admission. Age, drinking history, BMI, and gender were included as covariates. Statistical analyses employed both crude and adjusted GEE models with gender subgroup analysis.

**Results:**

Elevated CRP levels significantly correlated with increased AST (OR = 2.17, 95% CI: 1.14–4.12, *p* = 0.019) and ALT (OR = 2.37, 95% CI: 1.22–4.63, *p* = 0.011). The 72 h time point independently predicted ALT elevation (OR = 2.24, 95% CI: 1.37–3.65, *p* < 0.001). NLR (OR = 0.94, 95% CI: 0.90–0.99, *p* = 0.014) and WBC count (OR = 1.89, 95% CI: 1.12–3.18, *p* = 0.017) significantly predicted GGT elevation. Gender-stratified analysis revealed distinct patterns: In females, CRP elevation (both ≥40 mg/L and 10–40 mg/L) was inversely associated with AST (*p* < 0.05), and higher WBC correlated with lower GGT (*B* = –0.636, *p* = 0.017). In males, higher WBC was associated with lower AST; ALT decreased at 24 h (*B* = –1.08, *p* < 0.001) and 72 h (*B* = –1.11, *p* < 0.001); CRP 10–40 mg/L inversely correlated with ALT (*B* = –0.882, *p* = 0.008); and both higher WBC (*B* = –0.812, *p* = 0.013) and NLR (*B* = –0.063, *p* = 0.013) predicted lower GGT.

**Conclusion:**

CRP and white blood cell count significantly correlate with liver function changes in multiple injury patients, with marked gender-specific differences in these associations. These findings suggest the need for sex-specific monitoring strategies in trauma-related hepatic dysfunction.

## 1 Introduction

Multiple trauma is a clinical emergency that significantly threatens life and health, occurring in various scenarios such as traffic accidents, industrial incidents, and natural disasters. Despite advancements in medical technology that have markedly improved the survival rates of patients with severe trauma, complications—particularly organ failure—continue to adversely affect patient outcomes (1). The liver, a crucial organ for metabolism and detoxification, plays a significant role in the clinical outcomes of patients with multiple trauma (2–4). Impaired liver function disrupts metabolic balance and drug metabolism, potentially leading to adverse effects like coagulation disorders (5) and immune dysfunction (6). These complications can worsen the patient’s condition, prolong hospitalization, and increase medical costs. Therefore, understanding the mechanisms behind liver function impairment in patients with multiple trauma is theoretically and practically important for optimizing clinical management and enhancing patient outcomes.

In recent years, the inflammatory response has been recognized as a key factor contributing to liver damage in patients with multiple injuries (7, 8). Following trauma, the body rapidly initiates an acute inflammatory response to address tissue damage and mitigate infection risks (9). Inflammatory mediators, such as C-reactive protein (CRP) and white blood cell count (WBC), increase significantly during this process, reflecting the body’s stress state and immune response (10). However, an excessive inflammatory response may lead to systemic inflammatory response syndrome (SIRS), resulting in multiple organ dysfunction, including the liver (11). Studies have indicated a correlation between elevated CRP levels and increased liver enzymes (AST, ALT), suggesting that the acute inflammatory response may impact liver function through various pathways (12). Nevertheless, systematic studies examining the dynamic relationship between the inflammatory response and liver damage, as well as its influencing factors, remain relatively limited, particularly across different time points and subgroups.

Existing literature primarily focuses on single time-point studies or those with small sample sizes, lacking longitudinal analyses of changes in liver function among patients with multiple injuries at various stages post-trauma. Furthermore, gender, as a significant biological variable, may play a regulatory role in the relationship between inflammatory responses and liver damage (13); Differences in hormone levels, immune responses, and other factors between men and women may result in varying degrees of liver damage in response to identical inflammatory stimuli (14). However, systematic research on this topic remains insufficient and warrants further investigation.

This study aims to explore the association between liver function indicators (AST, ALT, GGT) and inflammatory markers (CRP, white blood cell count) in patients with multiple injuries at various time points post-trauma. Utilizing a repeated measurement cross-sectional design and a generalized estimating equation (GEE) model, the research will also assess the effects of drinking history, BMI, gender, age, and injury severity on this association. Through comprehensive data analysis, this study seeks to elucidate the dynamic relationship between inflammatory responses and liver function impairment, thereby providing a scientific foundation for early clinical prediction and personalized intervention strategies.

## 2 Materials and methods

### 2.1 Research subjects and sample selection

This study included trauma patients admitted to the trauma center of a tertiary hospital in Zhejiang Province, China, from January 2023 to December 2024. The sample size was determined using the geesample package in R software, with a significance level of *α* = 0.05 and a statistical power of 80%. The calculation accounted for three repeated measurements, an intraclass correlation of *ρ* = 0.5, and a correlation coefficient of approximately 0.3. The results indicated that a minimum sample size of approximately 84 cases was required. Ultimately, 114 valid samples were included in the study.

Inclusion criteria: Patients aged ≥18 years. Diagnosed with multiple injuries upon admission, defined as damage to ≥2 major body parts during the same illness period. At least one injury was life-threatening.

Exclusion criteria: Previous history of Immune and liver disease. Long-term use of immunosuppressants or anti-inflammatory drugs. Patients who have recently or chronically taken hepatotoxic drugs. Pregnancy or lactation. Abnormal liver function resulting from other organ failures.

**Figure 1:**
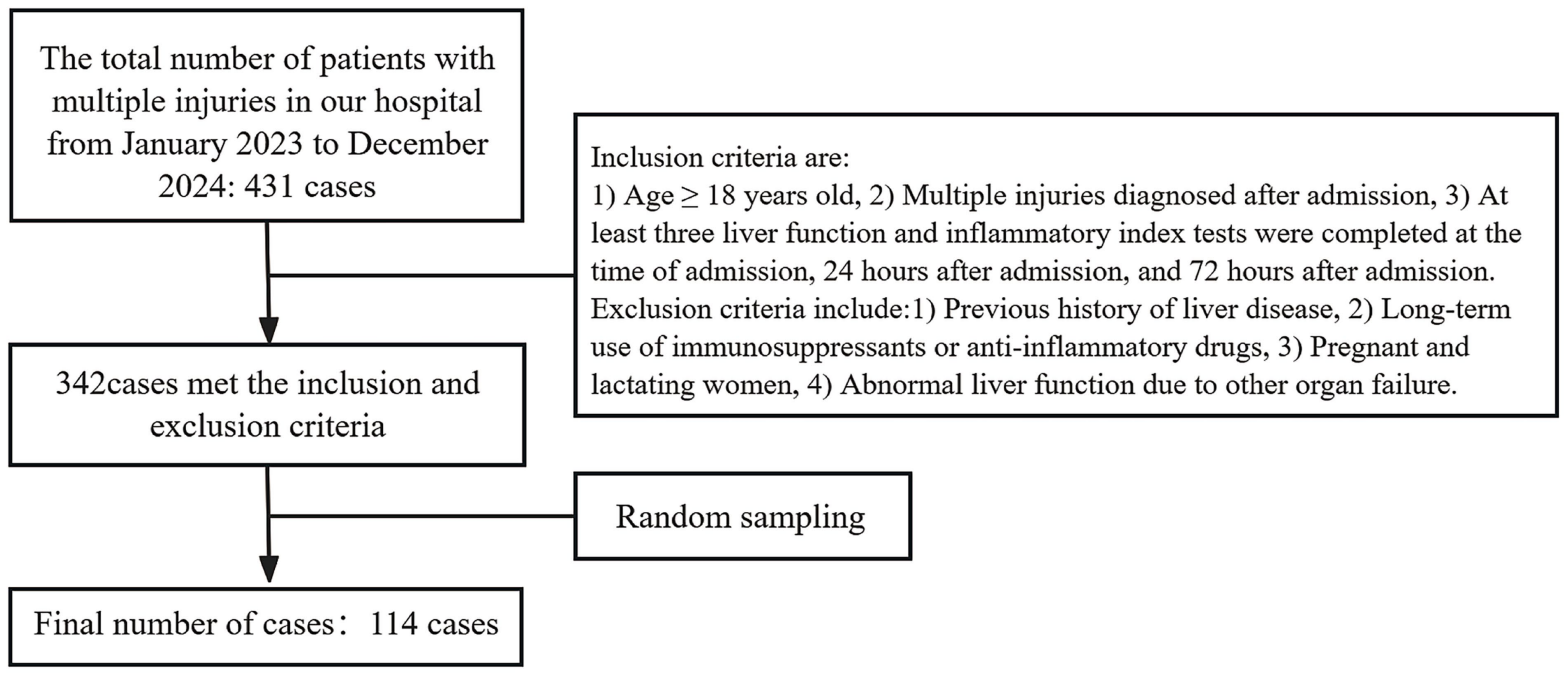
Flowchart of research subject selection.

A total of 431 patients with multiple injuries were admitted to the hospital over the two-year period. According to the established inclusion and exclusion criteria, a total of 342 cases were identified as meeting the requirements. Subsequently, simple random sampling was conducted based on the medical record numbers. Finally, 114 cases were selected for analysis, comprising 77 males and 37 females, with ages ranging from 18 to 85 years (Figure 1).

### 2.2 Data collection methods

All data were obtained from the hospital’s electronic medical record system to ensure integrity and accuracy. The primary variables collected included the Injury Severity Score (ISS), drinking history, BMI, age, gender, liver function indicators (AST, ALT, GGT), and inflammatory indicators (CRP, white blood cell count, NLR, LMR) measured at 0 h (admission), 24 h, and 72 h post-admission. Liver function and inflammatory indicators were assessed through blood tests conducted at these time points. The ISS was calculated based on six body regions: head and neck, face, chest, abdomen and pelvic organs, limbs and pelvis (excluding the spine), and body surface. The ISS value is the sum of the squares of the highest Abbreviated Injury Scale (AIS) scores in the three most severely injured regions.

### 2.3 Statistical analysis

All statistical analyses were performed using IBM SPSS Statistics version 27.0 for Windows. The point-line chart is drawn using the Windows version of oringin2021. Descriptive statistics were calculated for the basic characteristics of the subjects. Continuous variables were expressed as mean ± standard deviation, while categorical variables were presented as frequencies and percentages. Differences in categorical variables were assessed using chi-square tests; continuous variables with non-normal distributions were compared using nonparametric methods, while those following a normal distribution were analyzed by analysis of variance.

To explore the association between liver function indicators and inflammatory markers in patients with multiple injuries, a Generalized Estimating Equation (GEE) model (unstructured working-correlation matrix) was implemented for multivariate analysis, addressing repeated measurements and time series correlations in the data. Specifically, two models were developed:

Model 1 (Crude Model): Included the main independent variables—CRP, white blood cell count, ISS score, NLR, LMR, and time point.

Model 2 (Adjusted Model): Included all variables from Model 1 plus drinking history, BMI, gender and age as confounding variables.

The influence and statistical significance of each variable were assessed using the regression coefficient (B), odds ratio (OR), and 95% confidence interval (CI), with a significance threshold set at *p* < 0.05. Additionally, a gender subgroup analysis was performed by constructing separate GEE models for male and female patients to explore the moderating effect of gender on the relationship between liver function and inflammatory indicators. Statistical significance was determined using a two-tailed *p*-value of 0.05.

To evaluate the robustness of the model, AST, ALT, and GGT were remodeled in the following three ways: Changing the working correlation matrix from an unstructured matrix to an AP matrix; Changing age and BMI from categorical variables to continuous variables; excluding people aged 18 to 50 years old. Other model settings remain unchanged. Specific parameters of the sensitivity analysis are detailed in Supplementary Tables S1–S3.

### 2.4 Ethical statement

This study adheres to the ethical principles outlined in the Declaration of Helsinki. The research protocol received approval from the hospital ethics committee (ethical approval number: LS20241203). To ensure patient privacy, all clinical data collected during treatment were anonymized. As this study is a retrospective analysis, all participants provided informed consent upon admission, permitting the use of their clinical data for scientific research purposes.

### 2.5 Other necessary information

The data used in this research comply with relevant data protection laws and regulations. Upon publication of the paper, the research data will be made publicly available through the data sharing platform for other researchers to review and utilize.

## 3 Results

### 3.1 Baseline characteristics of participants

**Figure 2:**
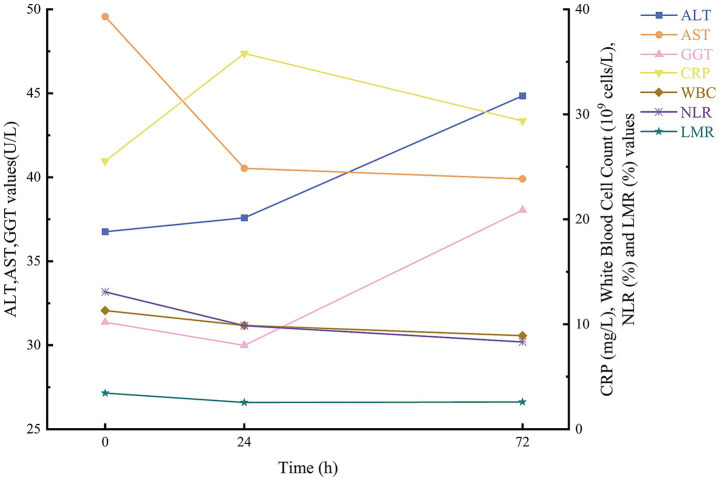
Trends of AST, ALT, GGT, CRP, WBC, NLR, and LMR over time.

In this study, 114 patients with multiple injuries were enrolled (Table 1) and stratified by Injury Severity Score (ISS) into three categories: mild-to-moderate (ISS < 16; *n* = 27), severe (16 ≤ ISS < 25; *n* = 31) and critical (ISS ≥ 25; *n* = 56). No significant differences in ALT or GGT levels were observed among these groups at any time point (all *p* > 0.05). By contrast, AST activity was markedly higher in patients with more severe injuries at 0 h (*p* = 0.011) and 24 h (*p* = 0.036). Inflammatory markers demonstrated a clear stepwise increase with injury severity: CRP concentrations differed significantly across groups at 0, 24, and 72 h (*p* < 0.001, p < 0.001 and *p* = 0.005, respectively), and white blood cell counts at 72 h were significantly elevated in the critical group (*p* = 0.037). The neutrophil-to-lymphocyte ratio (NLR) showed no baseline differences (*p* = 0.119) but rose in parallel with ISS at 24 h and 72 h (both *p* < 0.001). Conversely, the lymphocyte-to-monocyte ratio (LMR) declined progressively with increasing injury severity at 0 h (*p* = 0.005), 24 h (*p* < 0.001) and 72 h (*p* < 0.001). Temporal trends for AST, ALT, GGT, CRP, WBC, NLR and LMR are illustrated in Figure 2.

**Table 1:** Baseline characteristics.

Variable	Minor to Moderate Group(ISS < 16)	Serious Group (16 ≤ ISS < 25)	Severe Group (ISS ≥ 25)	F/H/χ^2^	***P***
*N*	27	31	56		
Age	52.260 ± 11.346	54.840 ± 13.011	57.960 ± 14.693	1.721	0.184
Gender					
Male	13	19	45	9.380	0.009*
Female	14	12	11		
BMI	24.052 ± 2.724	23.370 ± 2.785	23.930 ± 2.303	0.649	0.525
Drinking history					
No	16	24	35	2.644	0.267
Yes	11	7	21		
ALT					
0 h	32.452 ± 31.851	27.045 ± 15.883	44.202 ± 54.936	2.033	0.362
24 h	34.333 ± 24.667	29.423 ± 16.116	43.664 ± 51.111	0.366	0.833
72 h	51.822 ± 45.957	40.035 ± 24.92	44.152 ± 41.046	0.995	0.608
AST					
0 h	43.838 ± 71.656	30.072 ± 11.759	63.124 ± 79.063	8.964	0.011*
24 h	37.706 ± 32.433	27.598 ± 15.096	49.056 ± 59.035	6.674	0.036*
72 h	37.648 ± 30.242	32.897 ± 17.690	44.866 ± 45.927	0.374	0.829
GGT					
0 h	29.263 ± 28.785	23.746 ± 18.409	36.600 ± 45.547	1.105	0.575
24 h	29.126 ± 23.901	24.097 ± 17.650	33.663 ± 37.893	0.373	0.830
72 h	50.059 ± 61.158	29.913 ± 22.526	36.759 ± 41.323	2.698	0.260
CRP					
0 h	17.395 ± 39.34	16.85 ± 18.012	34.292 ± 32.323	18.252	<0.001*
24 h	28.492 ± 49.798	19.165 ± 21.345	48.533 ± 48.360	17.661	<0.001*
72 h	23.534 ± 33.553	20.108 ± 35.763	37.324 ± 48.471	10.793	0.005*
White Blood Cell Count					
0 h	10.141 ± 3.539	10.997 ± 3.984	12.030 ± 4.676	2.867	0.239
24 h	8.893 ± 3.247	9.709 ± 3.409	10.452 ± 3.468	3.738	0.154
72 h	7.885 ± 1.854	8.387 ± 2.374	9.695 ± 3.401	6.610	0.037*
NLR					
0 h	10.633 ± 7.887	12.183 ± 7.403	14.754 ± 11.451	4.249	0.119
24 h	7.428 ± 6.139	6.827 ± 5.798	12.699 ± 10.048	15.681	<0.001*
72 h	4.812 ± 3.559	5.098 ± 3.208	11.779 ± 11.671	20.6	<0.001*
LMR					
0 h	3.058 ± 3.511	6.088 ± 11.157	2.152 ± 1.415	10.631	0.005*
24 h	2.662 ± 1.430	3.434 ± 3.340	2.004 ± 1.596	16.556	<0.001*
72 h	3.250 ± 2.028	2.897 ± 1.256	2.101 ± 1.197	15.068	<0.001*

*P < 0.05.

### 3.2 Association between inflammatory markers and AST

We applied generalized estimating equations to model changes in AST expression over time, using the 0 h AST level as the reference (Table 2). In the unadjusted Model 1, CRP ≥ 40 mg/L was associated with a more than twofold increase in the odds of elevated AST (*B* = 0.773; OR = 2.17; 95% CI, 1.14–4.12; *p* = 0.019), whereas CRP 10–40 mg/L showed no significant association (*B* = 0.326; OR = 1.39; 95% CI, 0.88–2.19; *p* = 0.163). Neither elevated white blood cell count (>10 × 10^9^/L; B = 0.198; *p* = 0.284), nor ISS category (16–24 or ≥ 25; both *p* > 0.4), nor the 24 h and 72 h time points reached statistical significance. In Model 2, after further adjustment for drinking history, gender, age and BMI, CRP ≥ 40 mg/L remained the only independent predictor of AST elevation (*B* = 0.788; OR = 2.20; 95% CI, 1.15–4.20; *p* = 0.017), while CRP 10–40 mg/L again failed to achieve significance (*B* = 0.302; *p* = 0.200). No other covariates—including time, ISS, WBC, neutrophil–lymphocyte ratio, lymphocyte–monocyte ratio, BMI, drinking history, sex or age group—were significantly associated with AST elevation.

**Table 2:** Generalized estimating equation analysis of influencing factors of AST.

Variable	*B*	*P*	OR (95%CI)	*B*	*P*	OR (95%CI)
Intercept	−0.986	0.01*	0.373 (0.176,0.791)	−0.931	0.066	0.394 (0.146,1.065)
Time						
72 h	−0.119	0.638	0.888 (0.541,1.457)	−0.131	0.608	0.877 (0.532,1.446)
24 h	−0.431	0.068	0.650 (0.409,1.033)	−0.444	0.062	0.641 (0.402,1.022)
0 h						
ISS score						
≥25	0.315	0.436	1.371 (0.620,3.032)	0.275	0.537	1.316 (0.550,3.148)
16 ~ 25	0.303	0.495	1.354 (0.567,3.232)	0.247	0.575	1.281 (0.539,3.044)
<16						
White blood cell count						
>10	0.198	0.284	1.219 (0.849,1.750)	0.231	0.222	1.260 (0.870,1.826)
≤10						
CRP value						
≥40	0.773	0.019*	2.165 (1.138,4.120)	0.788	0.017*	2.200 (1.152,4.203)
10 ~ 40	0.326	0.163	1.385 (0.876,2.190)	0.302	0.200	1.352 (0.852,2.146)
<10						
NLR	0.004	0.808	1.004 (0.975,1.033)	0.001	0.934	1.001 (0.971,1.032)
LMR	−0.046	0.126	0.955 (0.901,1.013)	−0.043	0.128	0.958 (0.906,1.013)
Drinking history						
Yes				−0.360	0.325	0.698 (0.341,1.428)
No						
Gender						
Male				0.315	0.380	1.370 (0.678,2.770)
Female						
Age						
≥60				0.022	0.957	1.022 (0.458,2.282)
50 ~ 60				0.099	0.810	1.104 (0.493,2.472)
18 ~ 50						
BMI						
17 ~ 24				−0.407	0.256	0.666 (0.330,1.344)
>24						

*P < 0.05, Dependent variable: AST (<35 for normal group, ≥35 for high expression group, with normal as reference).

### 3.3 Association between inflammatory markers and ALT

Generalized estimating equations were applied to model ALT expression (normal vs. high) over time, with ALT at 0 h as the reference (Table 3). In the unadjusted Model 1, the odds of high ALT at 72 h were more than doubled compared with baseline (OR = 2.300; 95% CI 1.413–3.745; *p* = 0.001), and CRP ≥ 40 mg/L likewise predicted elevated ALT (OR = 2.404; 95% CI 1.245–4.641; *p* = 0.009). By contrast, the 24 h time point (OR = 1.113; *p* = 0.641), White Blood Cell Count > 10 × 10^9^/L (OR = 0.851; *p* = 0.484), ISS strata (16–25: OR = 1.187; *p* = 0.706; ≥ 25: OR = 1.174; *p* = 0.697), CRP 10–40 mg/L (OR = 1.007; *p* = 0.980), neutrophil–lymphocyte ratio (OR = 0.988; *p* = 0.496), lymphocyte–monocyte ratio (OR = 1.005; *p* = 0.783), BMI > 24 kg/m^2 (OR = 0.587; *p* = 0.193), drinking history and gender all failed to reach significance. In Model 2, further adjusted for drinking history, sex, age and BMI, the associations at 72 h (OR = 2.397; 95% CI 1.440–3.989; *p* = 0.001) and CRP ≥ 40 mg/L (OR = 2.728; 95% CI 1.391–5.350; *p* = 0.004) remained robust. Moreover, age ≥ 60 years emerged as a significant negative predictor of high ALT (OR = 0.370; 95% CI 0.163–0.842; *p* = 0.018). No other covariates—including the 24 h time point, ISS categories, WBC, CRP 10–40 mg/L, NLR, LMR, BMI > 24, drinking history or sex—were independently associated with ALT elevation.

**Table 3:** Generalized estimating equation analysis of factors affecting ALT.

Variable	*B*	*P*	OR(95%CI)	B	*P*	OR(95%CI)
Intercept	−1.488	<0.001*	0.226 (0.105,0.486)	−1.421	0.008*	0.242 (0.085,0.689)
Time						
72 h	0.806	0.001*	2.239 (1.373,3.649)	0.871	0.001*	2.39 (1.438,3.972)
24 h	0.107	0.641	1.113 (0.709,1.749)	0.132	0.574	1.141 (0.719,1.811)
0 h						
ISS score						
≥25	0.161	0.697	1.174 (0.522,2.641)	0.436	0.331	1.547 (0.642,3.731)
16 ~ 25	0.171	0.706	1.187 (0.486,2.896)	0.346	0.469	1.413 (0.555,3.6)
<16						
White blood cell count						
>10	−0.161	0.484	0.851 (0.543,1.336)	−0.203	0.377	0.816 (0.52,1.281)
≤10						
CRP value						
≥40	0.864	0.011*	2.373 (1.215,4.632)	1.071	0.002*	2.917 (1.459,5.834)
10 ~ 40	0.007	0.980	1.007 (0.585,1.735)	0.075	0.792	1.078 (0.619,1.876)
<10						
NLR	−0.012	0.496	0.988 (0.954,1.023)	−0.005	0.743	0.995 (0.963,1.028)
LMR	0.005	0.783	1.005 (0.969,1.043)	0	0.987	1 (0.964,1.036)
Drinking history						
Yes				0.161	0.667	1.175 (0.564,2.446)
No						
Gender						
Male				0.306	0.470	1.357 (0.593,3.107)
Female						
Age						
≥60				−1.112	0.013*	0.329 (0.137,0.791)
50 ~ 60				−0.204	0.631	0.815 (0.354,1.877)
18 ~ 50						
BMI						
>24				−0.533	0.193	0.587 (0.263,1.308)
17 ~ 24						

*P < 0.05, Dependent variable: ALT (<40 for normal group, ≥40 for high expression group, with normal as reference).

### 3.4 Association between inflammatory markers and GGT

Generalized estimating equations were applied to model GGT expression (normal vs. high) over time, with GGT at 0 h as the reference (Table 4). In the unadjusted Model 1, a white blood cell count >10 × 10^9^/L was associated with increased odds of high GGT (B = 0.524; OR = 1.689; 95% CI, 1.007–2.832; *p* = 0.047). Conversely, each unit increase in the neutrophil–lymphocyte ratio (NLR) predicted a modest reduction in GGT elevation (*B* = −0.057; OR = 0.944; 95% CI, 0.902–0.989; *p* = 0.014), and a BMI > 24 kg/m^2^ was inversely associated with high GGT (*B* = −1.046; OR = 0.352; 95% CI, 0.145–0.855; *p* = 0.021). Time point (24 h or 72 h), ISS category and CRP group did not reach statistical significance. Model 2, further adjusted for gender and age, confirmed White Blood Cell Count (>10 × 10^9^/L; *B* = 0.542; OR = 1.720; 95% CI, 1.030–2.873; *p* = 0.038) and NLR (*B* = −0.051; OR = 0.951; 95% CI, 0.907–0.996; *p* = 0.032) as independent predictors of high GGT. Moreover, CRP ≥ 40 mg/L emerged as a significant risk factor after adjustment (*B* = 0.680; OR = 1.975; 95% CI, 1.040–3.748; *p* = 0.037). No significant associations were observed for time point, ISS, LMR, drinking history or sex in either model.

**Table 4:** Baseline generalized estimating equation analysis of factors affecting GGT.

Variable	Model 1			Model 2		
	***B***	***P***	OR95%CI	*B*	***P***	OR95%CI
Intercept	−1.253	0.02*	0.286 (0.099,0.823)	−0.911	0.193	0.402 (0.102,1.587)
Time						
72 h	0.154	0.572	1.167 (0.684,1.99)	0.213	0.463	1.237 (0.701,2.183)
24 h	−0.067	0.779	0.936 (0.588,1.489)	−0.071	0.776	0.932 (0.572,1.517)
0 h						
ISS score						
≥25	0.103	0.837	1.108 (0.417,2.944)	0.466	0.410	1.594 (0.526,4.824)
16 ~ 25	−0.337	0.577	0.714 (0.219,2.332)	−0.054	0.933	0.947 (0.269,3.332)
<16		.				
White blood cell count						
>10	0.636	0.017*	1.889 (1.121,3.184)	0.740	0.011*	2.095 (1.187,3.697)
≤10					.	
CRP value						
≥40	0.505	0.097	1.657 (0.912,3.011)	0.680	0.037*	1.975 (1.04,3.748)
10 ~ 40	0.182	0.531	1.199 (0.68,2.116)	0.256	0.393	1.291 (0.718,2.322)
<10						
NLR	−0.057	0.014*	0.944 (0.902,0.989)	−0.051	0.032*	0.951 (0.907,0.996)
LMR	−0.093	0.256	0.911 (0.776,1.07)	−0.116	0.212	0.891 (0.742,1.068)
Drinking history						
Yes				0.728	0.114	2.07 (0.84,5.104)
No						
Gender						
Male				−0.588	0.255	0.556 (0.202,1.527)
Female						
Age						
≥60				−0.878	0.115	0.416 (0.139,1.239)
50 ~ 60				−0.052	0.914	0.949 (0.368,2.45)
18 ~ 50						
BMI						
>24				−1.046	0.021*	0.352 (0.145,0.855)
17 ~ 24						

*P < 0.05, Dependent variable: GGT (<50 for normal group, ≥50 for high expression group, with normal as reference).

### 3.5 Subgroup analysis

Sex-stratified GEE analyses (Model 1; Table 5) demonstrated distinct patterns of liver-enzyme regulation in male and female polytrauma patients. In male patients, White Blood Cell Count (>10 × 10^9^/L) was significantly associated with reduced AST expression (*B* = −0.423; *p* = 0.034). ALT levels fell markedly at both 24 h (*B* = −1.078; *p* < 0.001) and 72 h (*B* = −1.108; *p* < 0.001) relative to admission, and a CRP concentration of 10–40 mg/L predicted lower ALT (*B* = −0.882; *p* = 0.008). For GGT, both White Blood Cell Count (*B* = −0.812; *p* = 0.013) and a higher neutrophil–lymphocyte ratio (per unit increase; *B* = −0.063; *p* = 0.013) were significantly linked to decreased expression. No other covariates—including ISS category, CRP ≥ 40 mg/L, lymphocyte–monocyte ratio, BMI or time point effects on AST and GGT—reached significance. In female patients, AST expression was significantly lower at 24 h (*B* = −0.698; *p* = 0.001) and 72 h (*B* = −0.806; *p* = 0.001) versus baseline, and was inversely correlated with CRP levels of 10–40 mg/L (*B* = −0.857; *p* = 0.004) and ≥ 40 mg/L (*B* = −0.864; *p* = 0.011). ALT was also reduced in the CRP ≥ 40 mg/L subgroup (*B* = −0.773; *p* = 0.019). GGT expression diminished significantly in the presence of White Blood Cell Count (*B* = −0.636; *p* = 0.017) and with increasing neutrophil–lymphocyte ratio (*B* = −0.057; *p* = 0.014). ISS, lymphocyte–monocyte ratio, BMI and time-point effects on ALT and GGT did not achieve statistical significance in women.

**Table 5:** Generalized estimating equation analysis of influencing factors of AST, ALT and GGT by gender.

	Variable	AST		ALT		GGT	
		***B***	***P***	***B***	***P***	***B***	***P***
Male	Intercept	0.286	0.536	0.53	0.31	0.443	0.467
	Time						
	72 h	0.007	0.983	−1.108	<0.001*	−0.345	0.308
	24 h	−0.308	0.192	−1.078	<0.001*	−0.297	0.271
	0 h						
	ISS score						
	≥25	0.016	0.979	0.343	0.486	0.674	0.291
	16 ~ 25	−0.07	0.885	0.155	0.76	−0.681	0.369
	<16						
	White blood cell count						
	>10	−0.423	0.034*	−0.198	0.449	−0.812	0.013*
	≤10						
	CRP value						
	≥40	−0.404	0.283	−0.766	0.053	−0.236	0.451
	10 ~ 40	−0.323	0.212	−0.882	0.008*	−0.344	0.219
	<10						
	NLR	0.005	0.741	−0.015	0.513	−0.063	0.013*
	LMR	−0.106	0.054	−0.003	0.878	−0.22	0.087
Female	Intercept	0.182	0.675	0.181	0.634	0.145	0.791
	Time						
	72 h	−0.806	0.001*	0.119	0.638	−0.154	0.572
	24 h	−0.698	0.001*	−0.312	0.105	−0.221	0.354
	0 h						
	ISS score						
	≥25	−0.161	0.697	−0.315	0.436	−0.103	0.837
	16 ~ 25	0.011	0.979	−0.013	0.973	−0.439	0.424
	<16						
	White blood cell count						
	>10	0.161	0.484	−0.198	0.284	−0.636	0.017*
	≤10						
	CRP value						
	≥40	−0.864	0.011*	−0.773	0.019*	−0.505	0.097
	10 ~ 40	−0.857	0.004*	−0.447	0.07*	−0.324	0.245
	<10						
	NLR	−0.012	0.496	0.004	0.808	−0.057	0.014*
	LMR	0.005	0.783	−0.046	0.126	−0.093	0.256

*p < 0.05, the grouping of the dependent variables AST, ALT and GGT is the same as in Tables 2–4.

### 3.6 Sensitivity analysis

In sensitivity analyses, we assessed the robustness of our fully adjusted GEE model under three alternative specifications. First, replacing the unstructured correlation matrix with an autoregressive (AR) structure yielded effect estimates and *p* values for CRP (≥40 mg/L), WBC (>10 × 10^9/L) and liver enzymes (AST, ALT, GGT) that were virtually identical to those of the primary analysis. Second, exclusion of all participants aged 18–50 years (*n* = 42) did not materially alter the results, with p values for the key CRP–AST/ALT and WBC–GGT associations remaining below 0.05. Third, when age and BMI were modelled as continuous rather than categorical covariates, the direction, magnitude and statistical significance of these associations were preserved. Collectively, these sensitivity checks confirm the robustness of our model findings. For details, see Supplementary Tables S1–S3.

## 4 Discussion

In this longitudinal study of 114 polytrauma patients (342 enzyme measurements), we applied generalized estimating equations to examine associations between liver-function markers (AST, ALT, GGT) and inflammatory indices (CRP, white blood cell count, etc.) across sequential post-trauma time points. After adjusting for drinking history, age, sex, BMI, injury severity and other covariates, a CRP level ≥ 40 mg/L emerged as a strong independent predictor of both AST and ALT elevations, whereas white blood cell count (>10 × 10^9^/L) was significantly associated with increased GGT; moreover, the odds of elevated ALT more than doubled at 72 h compared with admission. Crucially, sex-stratified analyses revealed divergent response patterns: in men, both CRP ≥ 40 mg/L and the 72-h time point were linked to higher ALT levels, and elevated white blood cell count and neutrophil–lymphocyte ratio predicted GGT increases; in women, AST declined over time and was inversely correlated with CRP at 10–40 mg/L and ≥ 40 mg/L, ALT elevation was attenuated only in the CRP ≥ 40 mg/L subgroup, and GGT expression decreased in the presence of leukocytosis and rising NLR. These findings indicate that although systemic inflammation universally drives post-traumatic liver-enzyme alterations, the specific enzyme responses and temporal trajectories differ by sex, underscoring the need for gender-tailored monitoring and early intervention strategies.

Existing studies generally suggest that following multiple injuries, the body undergoes a stress inflammatory response characterized by a significant increase in cytokines, acute phase reaction proteins (such as CRP), and other mediators, which may lead to liver damage (15). Sadeghi et al.’s study demonstrated that in COVID-19 patients, C-reactive protein (CRP) levels were significantly elevated in the liver injury group, and CRP showed a significant correlation with aspartate aminotransferase (AST) and alanine aminotransferase (ALT) (16). This finding aligns with our results indicating that CRP values ≥40 were associated with abnormally high AST and ALT levels. As a prototypical acute phase response protein, CRP production is regulated by the liver’s synthetic capacity (17) and by cytokines such as interleukin-1 (IL-1) and interleukin-6 (IL-6) (18, 19). Following trauma, substantial tissue damage and cellular destruction rapidly activate the immune-inflammatory network, leading to a sharp increase in CRP levels within a short timeframe. As an indicator of injury status, CRP can exacerbate local inflammatory responses through the complement pathway or by binding to dead cells and pathogens, thereby placing additional stress on the liver (18). The white blood cell count serves as a reflection of the systemic inflammation activation level following trauma (20). Excessive inflammation or a cytokine storm can result in stress-induced hepatocyte damage, impairing the liver’s repair capacity. A review by Thakur S and colleagues on biomarkers of hepatotoxicity showed that elevated white blood cell counts generally indicate a higher risk of organ dysfunction (21). This observation is consistent with our findings that white blood cell counts >10 were significantly associated with increased gamma-glutamyl transferase (GGT) expression.

In this study, the increase in C-reactive protein (CRP) levels was closely associated with elevated liver function indicators, specifically aspartate aminotransferase (AST) and alanine aminotransferase (ALT), but not with gamma-glutamyl transferase (GGT). Conversely, the increase in white blood cell count (WBC) was significantly associated with high GGT levels, but not with AST and ALT. This discrepancy may arise from the distinct roles of these biomarkers: CRP and AST/ALT primarily reflect the inflammatory response and hepatocyte injury (22), whereas GGT is more indicative of bile metabolism (23) and oxidative stress processes (24). As an acute phase response protein, elevated CRP typically indicates the severity of systemic inflammation and hepatocyte injury. AST and ALT serve primarily to assess hepatocyte damage; when inflammation leads to hepatocyte necrosis or apoptosis, these enzymes are released into the bloodstream, resulting in increased levels. Thus, the correlation between CRP and AST/ALT is biologically plausible, suggesting direct hepatocyte damage due to the inflammatory response (25, 26). In contrast, GGT primarily reflects bile metabolism and the oxidative stress state of the liver. An increased WBC count suggests a more extensive inflammatory or infectious response within the body, which may impact the hepatobiliary system through oxidative stress and the release of pro-inflammatory cytokines, ultimately leading to elevated GGT levels. In addition, GGT is closely associated with factors such as alcohol intake, cholestasis, and drug-induced liver injury, which may explain the relationship between increased WBC count and GGT through various mechanisms (27). Existing studies further support the findings of this research. Smith et al. reported a positive correlation between CRP levels and AST and ALT in patients with acute liver injury; however, no significant association was found with GGT (28). Additionally, Yong-Jae Lee et al. found a positive correlation between white blood cell count and GGT levels in adults, which further strengthens the results of the present study (29). These findings illustrate the distinct association patterns of inflammatory indicators with various liver function parameters, indicating that CRP, WBC, AST, ALT, GGT, and other indicators should be evaluated comprehensively in clinical settings to gain a more thorough understanding of liver function status and the inflammatory response in trauma patients. This approach could guide personalized treatment strategies; for instance, patients exhibiting elevated CRP, AST, and ALT levels should receive timely anti-inflammatory interventions focused on liver cell damage, while those with increased WBC and GGT levels should be managed with a focus on bile metabolism and oxidative stress to prevent further deterioration of liver function.

The various manifestations arising from gender differences may be linked to the distinct biological effects of estrogen and androgen within the inflammatory regulation pathway. A review by Harding AT et al. indicates that estrogen influences the infection outcomes of individuals by modulating the host immune response. Specifically, estrogen can lower the levels of cytokines such as interferon-*γ* (IFN-γ), tumor necrosis factor-*α* (TNF-α), interleukin-1 (IL-1), and interleukin-6 (IL-6), thereby mitigating tissue damage and the incidence of chronic inflammation (30). The effects of estrogen are primarily mediated through estrogen receptors (ER), notably ERα and ERβ. In the synovial tissue of patients with rheumatoid arthritis, estrogen can regulate the activity of macrophages and fibroblasts, which in turn affects the inflammatory response (31). During severe trauma, the body’s hormonal regulation may become increasingly unbalanced or compensatory (32), leading to heightened sensitivity in female patients to specific indicators such as AST or ALT (33). In contrast, elevated androgen levels in men may enhance certain inflammatory signaling pathways and play a complex role in the tissue repair processes of skeletal muscle and liver (34). Rebecca J A et al. (35) reviewed the functions of androgens in relation to immune cells. Under the influence of androgens, innate immune cells (such as neutrophils, monocytes, and macrophages) increase the production of the anti-inflammatory cytokine IL-10 while decreasing the production of nitric oxide. Androgens also promote the differentiation of T cell subsets and reduce the production of inflammatory mediators (such as IFNG, IL-4, and IL-5). Additionally, androgens, including testosterone, can enhance the activity of gamma-glutamyl transferase (GGT). Ripple MO et al. (36) found that androgens can elevate the activity of GGT in human prostate cancer cells, as well as increase the mRNA and protein levels of GGT. Conversely, estrogens typically inhibit the increase of GGT (37). Collectively, these factors contribute to the observed differences in inflammatory indicators and patterns of liver function damage across different gender subgroups in this study. Consequently, it can be inferred that there may be an interactive effect between the body’s immune imbalance following multiple injuries and the influence of gender hormones, which significantly impacts patient prognosis.

This study presents notable innovations and practical significance in both method design and sample collection. Firstly, we employed repeated measurements to dynamically observe patients at various time intervals following trauma (specifically at 0, 24, and 72 h). This approach allows the analysis to more accurately reflect the continuous changes in liver function damage during the early and middle stages, rather than relying on a cross-sectional assessment at a single time point. Secondly, we controlled for multiple confounding factors using generalized estimating equations, constructing and comparing models for female and male patients. This method enhances our ability to elucidate the influence patterns under gender stratification. In contrast to traditional studies that typically conduct a single test at baseline or outcome, our longitudinal design not only captures more explanatory dynamic information but also provides a more robust reference for clinical practice in developing targeted intervention plans at different time intervals.

While this study has yielded certain findings, it is important to acknowledge its limitations. First, the study employs a single-center design, which may introduce selection biases regarding patient injury categories and treatment plans, thus limiting its external validity; therefore, a multi-center, large-sample prospective cohort study is required for future verification. Secondly, the study lacks a comprehensive evaluation and control of potential influencing factors, such as patients’ nutritional status, genetic polymorphisms, and specific drug regimens, making it challenging to eliminate the impact of these factors on the relationship between inflammation and liver function indicators. Additionally, our focus has primarily been on more common inflammatory indicators, such as C-reactive protein (CRP) and white blood cell counts, while other more sensitive or specific inflammatory factors, such as tumor necrosis factor-alpha (TNF-*α*) and interleukin-6 (IL-6), have not been included, resulting in deficiencies in the interpretation of the inflammatory network mechanism. As upstream cytokines, IL-6 and TNF-α both initiate the acute-phase response and can markedly influence ALT, AST, and GGT expression; their dynamic changes may occur before alterations in CRP or leukocyte count and exert important regulatory effects. By excluding these cytokines from the analysis, we may have underestimated or delayed the detection of early liver-enzyme elevations, and we were unable to assess their potential mediating or modulatory roles in liver injury pathways. Finally, the liver function classification indicators utilized in this study (aspartate aminotransferase (AST), alanine aminotransferase (ALT), and gamma-glutamyl transferase (GGT) categorized as ‘normal’ or ‘high expression’) are clinically feasible; however, they may not fully capture the extent or nature of liver damage. Future studies that incorporate additional key indicators, such as liver CT imaging changes and liver metabolite levels, to assess liver function status from multiple dimensions may enhance the accuracy and persuasiveness of the results.

The results of this study offer significant insights for the clinical practice of the trauma department. First, common detection indicators such as CRP and white blood cell count can serve as effective warning methods to swiftly assess the risk of liver damage in patients during the early stages. This capability enables the clinical team to implement timely and proactive intervention measures. For instance, in patients with severe trauma exhibiting extremely high CRP levels or markedly elevated white blood cell counts, it may be prudent to consider the administration of liver function protective medications, adjustments to fluid therapy, or enhanced monitoring protocols. Additionally, this study indicates that there are gender-based differences in the association patterns between CRP levels and liver damage. Consequently, clinical management processes can be tailored or refined for specific demographic groups, such as the early implementation of anti-inflammatory treatment plans or the enhancement of nutritional support, to mitigate the decline in liver function associated with the progression of inflammation. Overall, this study provides a robust foundation for early risk assessment and the optimization of personalized treatment strategies for patients with multiple injuries.

Based on existing findings, future research can be further developed in the following areas: First, expanding the sample size and conducting prospective cohort studies across multiple centers will enhance the representativeness and generalizability of the results. Second, a more comprehensive incorporation of potential confounding factors or intermediary variables, such as nutritional status, genetic polymorphisms, and cytokine profiles, is necessary to deeply analyze the physiological and pathological interactions between post-traumatic inflammation and liver function impairment. Third, integrating dynamic imaging and functional testing—such as ultrasound elastography, magnetic resonance spectroscopy analysis, and novel biomarkers—will allow for a multi-dimensional evaluation of liver damage and repair capacity. Fourth, the implementation of randomized controlled trials or intervention studies should be pursued to verify the efficacy of early anti-inflammatory strategies in patients of varying genders and injuries, while also exploring the effectiveness of individualized treatment plans aimed at improving prognosis and reducing hospital stays. Through these efforts, the pivotal role of the inflammatory response following multiple injuries in the progression of liver dysfunction can be more thoroughly elucidated, ultimately providing more scientific, accurate, and effective intervention measures for clinical practice and enhancing the recovery speed and long-term quality of life for patients with multiple injuries.

## 5 Conclusion

C-reactive protein (CRP) levels were significantly positively correlated with the expression of aspartate aminotransferase (AST) and alanine aminotransferase (ALT), while white blood cell counts exhibited a positive correlation with elevated gamma-glutamyl transferase (GGT) expression. These findings suggest that the inflammatory response plays a crucial role in liver function impairment. Furthermore, gender subgroup analysis revealed that in male trauma patients, both time and high CRP levels were significantly correlated with increased ALT expression, and white blood cell counts were associated with elevated GGT expression. In contrast, in the female cohort, high CRP levels were significantly correlated with increased AST expression. These results indicate that there are gender-specific differences in the impact of inflammation-related indicators on liver function damage in trauma patients. The findings of this study may assist clinicians in identifying high-risk individuals in the management of multiple trauma patients, particularly by monitoring CRP levels and white blood cell counts, and by implementing early intervention strategies tailored to different genders to improve liver function and overall prognosis.

## Data Availability

The raw data supporting the conclusions of this article will be made available by the authors, without undue reservation.
