# Supplementary material for: Inflammatory markers CRP and WBC as predictors of liver function impairment in multiple injury trauma patients: a repeated measures analysis
Source: Front Med (Lausanne). 2025 Aug 1;12:1570474. doi: 10.3389/fmed.2025.1570474 (PMC12354656; doi:10.3389/fmed.2025.1570474)
Supplement: Supplementary file 1 [file Table_1.docx]

Supplementary Table 1. The impact factors of AST, ALT and GGT in the calculation process used by AR squares

|  | ALT | | AST | | GGT | |
| --- | --- | --- | --- | --- | --- | --- |
| Variable | B | ***P*** | B | ***P*** | B | ***P*** |
| Intercept | -1.432 | 0.007 | -0.744 | 0.136 | -0.979 | 0.146 |
| Time | | | | | | |
| 72h | 0.878 | 0.001 | -0.158 | 0.537 | 0.196 | 0.494 |
| 24h | 0.138 | 0.557 | -0.458 | 0.054 | -0.092 | 0.713 |
| 0h |  |  |  |  |  |  |
| ISS Score | | | | | | |
| ≥25 | 0.443 | 0.324 | 0.173 | 0.686 | 0.468 | 0.409 |
| 16~25 | 0.346 | 0.471 | 0.104 | 0.808 | -0.043 | 0.947 |
| <16 |  |  |  |  |  |  |
| White Blood Cell Count | | | | | | |
| >10 | -0.221 | 0.339 | 0.201 | 0.294 | 0.774 | 0.007 |
| ≤10 |  |  |  |  |  |  |
| CRP Value | | | | | | |
| ≥40 | 1.089 | 0.002 | 0.767 | 0.014 | 0.775 | 0.028 |
| 10~40 | 0.087 | 0.762 | 0.22 | 0.331 | 0.249 | 0.432 |
| <10 |  |  |  |  |  |  |
| Drinking history | | | | | | |
| Yes | 0.17 | 0.65 | -0.41 | 0.256 | 0.765 | 0.093 |
| No |  |  |  |  |  |  |
| Gender | | | | | | |
| male | 0.281 | 0.506 | 0.394 | 0.271 | -0.607 | 0.235 |
| female |  |  |  |  |  |  |
| Age | | | | | | |
| ≥60 | -1.13 | 0.012 | -0.038 | 0.925 | -0.824 | 0.136 |
| 50~60 | -0.206 | 0.627 | 0.067 | 0.869 | -0.037 | 0.938 |
| 18~50 |  |  |  |  |  |  |
| BMI | | | | | | |
| >24 | -0.526 | 0.2 | -0.432 | 0.225 | -1.053 | 0.023 |
| 17~24 |  |  |  |  |  |  |
| NLR | -0.004 | 0.802 | -0.002 | 0.906 | -0.054 | 0.03 |
| LMR | 0.001 | 0.941 | -0.04 | 0.185 | -0.1 | 0.246 |

Supplementary Table 2. Generalized estimating equation analysis of influencing factors of AST, ALT and GGT after excluding people aged 18-50 years

|  | ALT | | AST | | GGT | |
| --- | --- | --- | --- | --- | --- | --- |
| Variable | B | ***P*** | B | ***P*** | B | ***P*** |
| Intercept | -1.923 | 0.004 | -0.707 | 0.16 | -0.96 | 0.108 |
| Time |  |  |  |  |  |  |
| 72h | 1.003 | 0.004 | -0.287 | 0.367 | -0.019 | 0.958 |
| 24h | 0.025 | 0.939 | -0.67 | 0.028 | -0.319 | 0.326 |
| 0h |  |  |  |  |  |  |
| ISS Score |  |  |  |  |  |  |
| ≥25 | 0.614 | 0.327 | 0.151 | 0.771 | 0.555 | 0.522 |
| 16~25 | 0.777 | 0.222 | 0.276 | 0.603 | 0.424 | 0.61 |
| <16 |  |  |  |  |  |  |
| White Blood Cell Count |  |  |  |  |  |  |
| >10 | -0.386 | 0.179 | 0.352 | 0.164 | 0.613 | 0.083 |
| ≤10 |  |  |  |  |  |  |
| CRP Value |  |  |  |  |  |  |
| ≥40 | 1.322 | 0.005 | 0.854 | 0.038 | 0.678 | 0.056 |
| 10~40 | 0.286 | 0.421 | 0.387 | 0.178 | 0.405 | 0.301 |
| <10 |  |  |  |  |  |  |
| Drinking history |  |  |  |  |  |  |
| Yes | -0.317 | 0.536 | -0.408 | 0.384 | 0.149 | 0.815 |
| No |  |  |  |  |  |  |
| Gender |  |  |  |  |  |  |
| male | 0.167 | 0.736 | 0.117 | 0.774 | -0.906 | 0.114 |
| female |  |  |  |  |  |  |
| Age |  |  |  |  |  |  |
| ≥60 | -0.801 | 0.101 | 0.059 | 0.889 | -0.748 | 0.222 |
| 50~60 |  |  |  |  |  |  |
| BMI |  |  |  |  |  |  |
| >24 | -0.147 | 0.762 | -0.219 | 0.618 | -0.552 | 0.247 |
| 17~24 |  |  |  |  |  |  |
| NLR | -0.009 | 0.574 | -0.003 | 0.893 | -0.039 | 0.137 |
| LMR | 0.011 | 0.53 | -0.059 | 0.138 | -0.087 | 0.095 |

Supplementary Table 3. Age and BMI were converted into continuous variables and generalized estimating equations were used to analyze the influencing factors of AST, ALT and GGT.

|  | ALT | | AST | | GGT | |
| --- | --- | --- | --- | --- | --- | --- |
| Variable | B | ***P*** | B | ***P*** | B | ***P*** |
| Intercept | 0.934 | 0.643 | -0.189 | 0.917 | 1.376 | 0.533 |
| Time |  |  |  |  |  |  |
| 72h | 0.862 | 0.001 | -0.124 | 0.626 | 0.177 | 0.527 |
| 24h | 0.134 | 0.571 | -0.435 | 0.066 | -0.078 | 0.748 |
| 0h |  |  |  |  |  |  |
| ISS Score |  |  |  |  |  |  |
| ≥25 | 0.345 | 0.433 | 0.261 | 0.546 | 0.342 | 0.543 |
| 16~25 | 0.317 | 0.506 | 0.215 | 0.626 | -0.12 | 0.854 |
| <16 |  |  |  |  |  |  |
| White Blood Cell Count |  |  |  |  |  |  |
| >10 | -0.254 | 0.289 | 0.189 | 0.31 | 0.665 | 0.017 |
| ≤10 |  |  |  |  |  |  |
| CRP Value |  |  |  |  |  |  |
| ≥40 | 1.052 | 0.003 | 0.781 | 0.017 | 0.666 | 0.043 |
| 10~40 | 0.082 | 0.772 | 0.314 | 0.184 | 0.225 | 0.461 |
| <10 |  |  |  |  |  |  |
| Drinking history |  |  |  |  |  |  |
| Yes | 0.259 | 0.499 | -0.331 | 0.364 | 0.851 | 0.057 |
| No |  |  |  |  |  |  |
| Gender |  |  |  |  |  |  |
| male | 0.065 | 0.88 | 0.218 | 0.547 | -0.691 | 0.212 |
| female |  |  |  |  |  |  |
|  |  |  |  |  |  |  |
| NLR | -0.005 | 0.773 | 0.004 | 0.814 | -0.053 | 0.025 |
| LMR | 0.002 | 0.931 | -0.047 | 0.145 | -0.095 | 0.262 |
| Age | -0.035 | 0.015 | -0.006 | 0.645 | -0.014 | 0.353 |
| BMI | -0.036 | 0.619 | -0.019 | 0.759 | -0.085 | 0.294 |
